# Assessment of Fertility Outcomes Following Combined Clomiphene and Letrozole Versus Letrozole Therapy for the Treatment of Polycystic Ovarian Syndrome Subfertility

**DOI:** 10.7759/cureus.38886

**Published:** 2023-05-11

**Authors:** Priyanka Sharma, Ramesh Chandra, Avir Sarkar, Sonam Jindal, Annu Sharma, Jagadish C Sharma, Saroj Jaggarwal

**Affiliations:** 1 Obstetrics and Gynecology, Employee State Insurance Corporation (ESIC) Medical College and Hospital, Faridabad, Faridabad, IND; 2 Obstetrics and Gynecology, Employee State Insurance Corporation (ESIC) Medical College and Hospital, Faridabad, IND

**Keywords:** ovulation induction, clomiphene citrate, letrozole, subfertility, polycystic ovarian syndrome

## Abstract

Background: Poly Cystic Ovarian Syndrome (PCOS) affects 8-13%^ ^of women in their reproductive age and is one of the foremost causes of female subfertility. Traditionally, clomiphene citrate has been the first-line treatment for ovulation induction in PCOS. However, the European Society of Human Reproduction and Embryology (ESHRE) international evidence-based guidelines in 2018 recommended the use of letrozole as first-line therapy for ovulation induction in anovulatory PCOS women, due to better pregnancy and live birth rates. Here we aimed to evaluate the effect of combined - clomiphene and letrozole versus letrozole for the treatment of PCOS subfertility.

Methods: It was a retrospective cohort study conducted on reproductive-age women fulfilling Rotterdam Criteria for PCOS with a history of subfertility. All participants receiving at least one cycle of letrozole and clomiphene combination were recruited as cases. However, women receiving letrozole only for ovulation induction were taken as controls. Hospital records were abstracted for data on baseline characteristics such as age, duration of infertility, PCOS phenotype, body mass index (BMI), past medical and fertility history, treatment with ovulation induction agents, and metformin use. The mean size of the largest follicle, number of dominant follicles of size greater than 15 mm and endometrial thickness on Days 12-14 or on the day of the luteinizing hormone (LH) surge were recorded. A cycle was termed ovulatory if serum progesterone levels were > 5.0 ng/ml on the seventh day after the LH surge or day 22 in the absence of the LH surge. Data pertaining to therapy-associated side effects were also abstracted from the clinical records.

Results: Amongst the ovulatory cycles in both groups, there was no significant difference in the day of the LH surge. Serum progesterone levels on the seventh day post-ovulation were higher with combination therapy (19.35 v/s 26.71, p=0.004). The number of ovulatory cycles was also greater with combination therapy, but the difference was just short of significant (25 vs 18, p=0.08). The mean diameter of the largest follicle, incidence of multi-follicular ovulation, and thin endometrium were similar in both groups. The adverse effect profile was similar in both groups.

Conclusion: Combination treatment of clomiphene citrate with letrozole may potentially improve fertility outcomes in PCOS subfertility in terms of the likelihood of ovulation and higher post-ovulatory progesterone levels, however, larger studies are required.

## Introduction

Poly Cystic Ovarian Syndrome (PCOS) affects 8-13% [1-4] of women in their reproductive age and is one of the foremost causes of female subfertility [5]. Treatment modalities focus on induction of ovulation in these women directly through the use of clomiphene citrate, aromatase inhibitors, gonadotrophins, or assisted reproduction protocols, or indirectly through weight loss, insulin sensitizers, and ovarian drilling, all of which improve the disordered hormonal milieu seen in PCOS. Traditionally, clomiphene citrate has been the first-line treatment for ovulation induction in PCOS. A selective estrogen receptor modulator, clomiphene citrate, through action on nuclear estrogen receptors, reduces negative estrogenic feedback on the pituitary and hypothalamus and increases gonadotrophin secretion, which accelerates follicular development [6]. Clomiphene treatment achieves an ovulation rate of 80%. However, due to its detrimental effect on the endometrium and cervical mucus consistency, pregnancy rates remain low at 40% [6,7].

The European Society of Human Reproduction and Embryology (ESHRE) International evidence-based guidelines in 2018 recommended the use of letrozole as first-line therapy for ovulation induction in anovulatory PCOS women due to better pregnancy and live birth rates compared to clomiphene citrate [8]. In contrast to clomiphene citrate, it promotes mono-follicular ovulation and does not adversely affect the endometrium [9]. Since both drugs have different mechanisms of action, it is plausible that combination therapy with both may yield better ovulation and pregnancy rates than either drug alone. In this study, we tried to compare the fertility outcomes of PCOS sub-fertile women receiving combined clomiphene and letrozole versus letrozole therapy for ovulation induction.

## Materials and methods

This retrospective cohort study conducted in the Department of Obstetrics and Gynecology at Employee State Insurance Corporation (ESIC) Medical College and Hospital, Faridabad, India, compared combination letrozole and clomiphene therapy with letrozole alone for ovulation induction in PCOS sub-fertile women from January 2020 to December 2022. Ethical approval was taken prior to the commencement of the study from the registered institutional ethical committee (134 X/11/13/2023- IEC/54).

**Figure 1:**
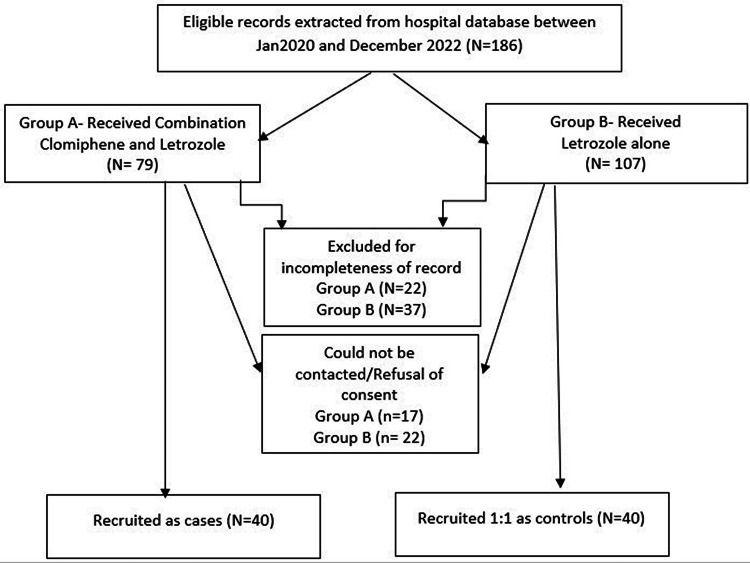
Schematic diagram depicting the recruitment process for the study

Reproductive age (21-40 years) women fulfilling the 2003 Rotterdam Criteria [10] for PCOS with a history of subfertility, who received at least one cycle of a combination of letrozole 2.5 mg and clomiphene citrate 50 mg from the third to the seventh day of their menstrual cycle for ovulation induction during the study period were recruited to the study cohort. An equal number of age-matched PCOS sub-fertile women who met all the defined eligibility criteria and had received at least one cycle of letrozole 2.5 mg from the third to the seventh day of the menstrual cycle for ovulation induction in the defined study period were recruited as controls for comparison (Figure 1). 

Informed consent was taken from all participants. Subfertility was defined as the inability of a couple to achieve pregnancy after 12 months of regular timed unprotected intercourse in women less than 35 years of age or after six months in women more than 35 years of age. Women with non-patent fallopian tubes (on hysterosalpingography or laparoscopic chromo-pertubation) were excluded. Couples with male factor subfertility (defined as total sperm count less than 15 million per ml or rapidly progressive sperm motility lesser than 32%) were excluded from the study [11]. Women with uncorrected and/or overt thyroid disease and hyperprolactinemia, Cushing’s syndrome, late-onset congenital adrenal hyperplasia and hormone-secreting adrenal and ovarian cancers were excluded from the analysis. If a patient received more than one cycle of ovulation induction treatment with either or both the regimens under evaluation, only the first cycle was included in the analysis. Those who had received either of the two treatments within the past three months were also excluded from the study.

Hospital records were abstracted for data on baseline characteristics such as age, duration of infertility, PCOS phenotype, BMI (Body Mass Index), past medical and fertility history, treatment with ovulation induction agents and metformin use. Oral glucose tolerance test (OGTT) and HbA1C values were also recorded. Serial follicular monitoring data were studied to note the number of dominant follicles recruited per cycle. Day of Luteinizing Hormone (LH) surge (positive urinary ovulation prediction kit) was noted for each cycle. The mean size of the largest follicle, number of dominant follicles of size greater than 15 mm, and endometrial thickness on Day 12-14 or on the day of LH surge were recorded. A cycle was termed ovulatory if serum progesterone levels were > 5 ng/ml on the seventh day after the LH surge or day 22 in the absence of the LH surge. Data pertaining to therapy-associated side effects were also abstracted from the clinical records.

All data were entered into an MS Excel Sheet and checked for completeness. Data was tested for normality with the Kolmogorov-Smirnov test. For categorical variables, the Chi-square test was used at a two-sided significance level of 0.05 for testing differences between the two treatment groups. For continuous variables, the mean + standard deviation in each group was reported and differences between groups were analyzed using an unpaired student t-test. A subgroup analysis was performed to assess follicular growth parameters and endometrial thickness in those who ovulated. Statistical analysis was done using SPSS Version 23.0 (IBM Corp., Armonk, NY)

## Results

The baseline characteristics of Group A (letrozole use) and Group B (combination clomiphene and letrozole use) are detailed in Table 1. There was no statistically significant difference in age, BMI, or the phenotypic features of PCOS such as the severity of oligo-and anovulation, or the presence of hyperandrogenism in the two groups.

**Table 1:** Clinical and biochemical characteristics of study groups *Expressed as Mean (Standard Deviation) BMI: Body Mass Index; OGTT: Oral Glucose Tolerance Test; HBA1C: Glycated Hemoglobin

Parameters	Group A (N=40) Letrozole Use	Group B (N=40) Clomiphene + Letrozole Use	P value
Mean Age (years)*	28.13 (3.68)	28.05 (4.45)	0.075
Mean BMI (kg/m^2^)*	25.16 (2.76)	25.21 (3.28)	0.947
Number of Menstrual cycles per year*	7.50 (1.73)	7.58 (1.63)	0.843
Oligomenorrhoea	33	34	0.788
Severe Oligomenorrhoea	14	14	
Hyperandrogenism	14	11	0.237
Primary Infertility	28	24	0.241
Secondary Infertility	12	16	
Previous Live Births	10	7	0.293
Mean duration of infertility (years)*	4.25 (2.03)	5.33 (3.08)	0.070
Clomiphene Ever-Use	7	10	0.498
Letrozole Ever-Use	16	8	0.043
Current Metformin Use	12	4	0.024
OGTT – 0 hours (mg/dl)*	83.02 (14.41)	81.95 (8.68)	0.687
OGTT- 2 hours (mg/dl)*	122.0 (15.97)	112.65 (12.2)	0.004
HBA1C (%)*	5.0 (0.73)	4.75 (0.57)	0.088

**Figure 2:**
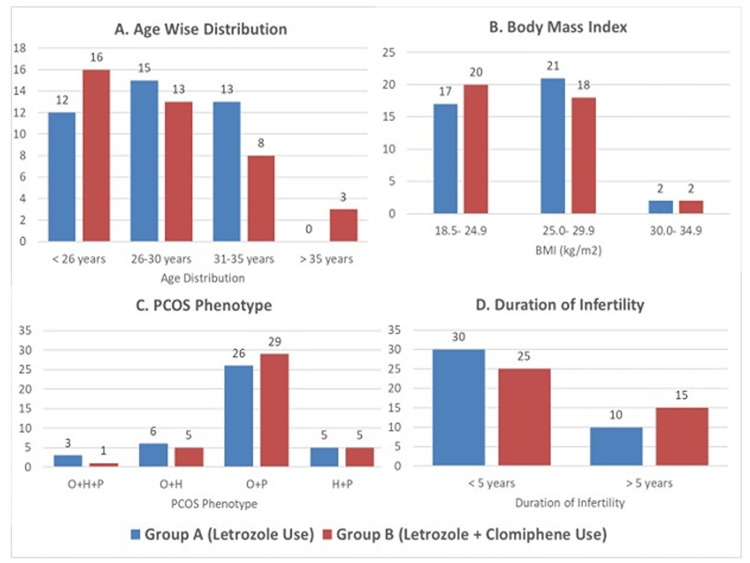
Distribution of baseline characteristics of the study population in both groups p-value of A=0.179; B=0.789; C=0.740; D=0.167 O stands for ovulatory, H for hyperandrogenism, and P for Polycystic ovarian morphology in ultrasound

The prevalence of primary and secondary subfertility and mean duration of fertility were similar in both groups. More women in Group A had received letrozole in the past than in Group B (16 v/s 8, p=0.043). However, there was no significant difference in the previous use of clomiphene for ovulation induction. A significantly greater number of women in Group A were taking metformin at the time of ovulation induction, compared to Group B (12 v/s 4, p=0.02). Two-hour OGTT levels were significantly higher in Group B (122.0 + 15.97 mg/dl v/s 112.65 + 12.2 mg/dl; p=0.004). Figure 2 reflects that both groups were similar with regard to key parameters that can impact response to ovarian stimulation.

Table 2 describes the pattern of ovulation observed in the study groups. Recruitment of dominant follicles was similar in both groups (22 v/s 25, p=0.05). There was no difference in mono-follicular or multi-follicular recruitment. The largest follicle size attained on Days 12 to 14 of the cycle or on the day of the LH surge detected by a urinary prediction kit was similar in both groups. LH Surge was observed in 19 cycles in Group A and 25 cycles in Group B (p=0.308). In Group A, 18 cycles were ovulatory (based on raised serum progesterone levels), as compared to 25 cycles in Group B. This difference was just short of significant (p=0.08). 

**Table 2:** Ovulatory function in the study groups * Expressed as Mean (Standard Deviation)

Ovulatory Parameters	Group A (N=40)	Group B (N=40)	P value
No of Dominant Follicle > 10 mm	22	25	0.928
One follicle	14	15	
Two follicles	8	15	
Three follicles	2	9	
No of Dominant Follicle > 15 mm	19	25	0.262
One follicle	13	21	
Two follicles	5	4	
Three follicles	1	0	
Largest Follicle size*	19.38 (1.89)	20.22 (2.62)	0.259
Luteinizing Hormone Surge detected	19	24	0.308
Ovulatory cycles	18	25	0.08

Amongst the ovulatory cycles in both groups (Table 3), there was no significant difference in the days of the LH surge (p=0.558). The mean diameter of the largest follicle was also similar (p=0.259). Both groups saw a similar prevalence of thin endometrium on Days 12 to 14 or on the day of LH surge, indicating that the addition of clomiphene to letrozole did not significantly compromise the endometrial growth and maturation. Mean serum progesterone levels measured on the seventh day post-LH surge were significantly higher in Group B compared to Group A (19.35 + 4.47 v/s 26.71 + 9.35; p=0.004). 

**Table 3:** Quality parameters for ovulatory cycles in study groups *Expressed as Mean (Standard Deviation) **ET = Endometrial Thickness

Ovulatory Parameters	Group A (N=18)*	Group B (N=25)*	P value
Mean Day of Luteinizing Hormone Surge	11.0 (1.71)	11.56 (3.74)	0.558
Largest Follicle Size (mm)	19.38 (1.89)	20.22 (2.62)	0.259
Thin ET** (< 6mm)	8.76 (1.27)	8.50 (1.30)	0.461
Mean serum Progesterone levels (ng/ml)	19.35 (4.47)	26.71 (9.35)	0.004

No significant difference was observed in the medication-related adverse effect profile among the groups (Table 4). No serious adverse event (ovarian hyperstimulation and/or treatment-related hospitalization) was observed in either group.

**Table 4:** Adverse effect profiles in study groups

Adverse Effect	Group A (N=40)	Group B (N=40)	P value
Headache	3	2	0.500
Nausea & Vomiting	5	2	0.216
Bloating	1	0	0.500
Fatigue	1	4	0.179
Backache	3	2	0.500
Breast Discomfort	8	3	0.960
Night Sweats	2	2	0.692

## Discussion

Ovulation induction forms the background for assisted fertility treatment in PCOS sub-fertile women. Clomiphene citrate was the time-tested inducing agent down the line for decades. Letrozole has now overtaken clomiphene as the first-line recommendation for ovulation induction. However, resistance to either agent is a significant challenge to fertility treatment, especially when assisted reproductive services are beyond the financial reach of many middle and low-income couples.

Letrozole has already proved beneficial in women with clomiphene resistance. A randomized controlled trial (RCT) conducted in 2019 concluded that there is a significant improvement in endometrial thickness (8.06 ±0.38 vs 7.2 ±0.61), ovulation rates (22.45 ± 2.21 vs 19.98 ± 1.88), pregnancy rates (81% vs 22% P=0.00) in women who received letrozole when compared to those who received clomiphene citrate [12]. However, in patients not responding to letrozole induction, clinicians are in a dilemma whether to adopt second-line therapeutic options like gonadotropins, especially in women who cannot afford costlier treatment modalities. A few studies have compared the fertility outcomes of adding clomiphene to letrozole therapy versus letrozole alone in women with PCOS [13,14].

Ibrahem et al. conducted an RCT on 60 clomiphene-resistant PCOS women randomized to two groups, the first receiving combined letrozole and clomiphene citrate and other receiving letrozole alone. It was noted that there was no significant difference in endometrial thickness, rate of ovulation, and clinical pregnancy, however, progesterone measured seven days after the expected time of ovulation in the letrozole group was observed to be higher than clomiphene citrate group [13]. Similarly, a recent United States based RCT on 70 sub-fertile PCOS women compared letrozole treatment alone with combination letrozole and clomiphene therapy [14]. The mid-luteal serum progesterone concentration greater than 3 mg/dl was a successful outcome measure. They concluded that combination therapy of letrozole and clomiphene was associated with a higher ovulation rate compared with letrozole alone as compared with combined therapy.

Hajishafiha et al. conducted a population-based survey on 100 patients of infertility who failed to ovulate alone with clomiphene citrate and letrozole as monotherapy, these patients underwent 257 cycles of a combination of letrozole and clomiphene in which 213 were able to form dominant follicle (82.9%), and 44 were unable to do so (17.1%) [15]. The mean endometrial thickness on the day of human chorionic gonadotropin administration was 8.17 ±1.3 mm and the pregnancy rate was 42%. It was concluded that in PCOS women resistant to clomiphene and letrozole individually, a combination of these two drugs could be used before using more aggressive treatment. These results bear special promise to our population where PCOS is rampant and rising, and treatment options are generally limited due to a multitude of factors including cost, time, and safety. Our institute, in particular, caters to women from low socioeconomic strata. Our data demonstrates a slight superiority of the combination of letrozole and clomiphene over letrozole alone in terms of improved ovulation rates and higher mean serum progesterone levels achieved in ovulatory cycles. There is no additional detrimental effect of the addition of clomiphene on endometrial development, which is reassuring. The side effect profile of both these groups is similar, which is reassuring. In these women, letrozole and clomiphene combination therapy would be an appropriate alternative before proceeding to costlier, lengthier, and riskier treatments such as gonadotrophin therapy or in vitro fertilization. A combination of clomiphene and letrozole therapy could also be considered a potential first-line treatment option for PCOS infertility, due to better ovulatory outcomes, which could be further confirmed with larger studies in randomized controlled settings.

This retrospective cohort study uses age and BMI-matched controls as external comparators, allowing for more accurate comparison by eliminating known confounders. This study is, however, limited by a small sample size. The difference in ovulation rates in each group, as well number of dominant follicles recruited was just short of significant. We believe that a larger sample could have potentially demonstrated a more concrete advantage of combination therapy in this aspect. In our study, mean two-hour OGTT levels were significantly higher in group A (letrozole alone), although they did not reach hyperglycemia cut-offs. Similarly, there was greater metformin use in the letrozole-only group. This could also account for the meagreness of the difference in ovulatory cycles, since the control group (Group A) could have, in fact, surreptitiously benefitted from the addition of metformin to the ovulation induction treatment. Also, long-term follow-ups such as clinical pregnancy rates and live birth rates could not be included due to a lack of data records and loss to follow-up due to the coronavirus disease 2019 (COVID-19) pandemic. The authors acknowledge that an RCT is the design of choice for testing treatment regimens. This study too was envisaged initially as a randomized controlled trial. However, the outbreak of the COVID-19 pandemic allowed only for a retrospective review of the available clinical data.

## Conclusions

Both letrozole and clomiphene have been used individually with modest success in achieving ovulation and pregnancy in PCOS women. Their effect and side effect profiles are well documented. Initial research indicates that combined letrozole and clomiphene therapy is showing promise in PCOS subfertile women with superior ovulation rates than the current standards of treatment. This novel approach using traditionally tested drugs thus has immense potential as a low-cost, low-risk, and time-saving alternative to further treatments such as gonadotrophins or in vitro fertilization.

## References

[1] Azziz R, Carmina E, Dewailly D (2006). Positions statement: criteria for defining polycystic ovary syndrome as a predominantly hyperandrogenic syndrome: an Androgen Excess Society guideline. J Clin Endocrinol Metab.

[2] Diamanti-Kandarakis E, Kandarakis H, Legro RS (2006). The role of genes and environment in the etiology of PCOS. Endocrine.

[3] March WA, Moore VM, Willson KJ, Phillips DI, Norman RJ, Davies MJ (2010). The prevalence of polycystic ovary syndrome in a community sample assessed under contrasting diagnostic criteria. Hum Reprod.

[4] Bozdag G, Mumusoglu S, Zengin D, Karabulut E, Yildiz BO (2016). The prevalence and phenotypic features of polycystic ovary syndrome: a systematic review and meta-analysis. Hum Reprod.

[5] Azziz R, Woods KS, Reyna R, Key TJ, Knochenhauer ES, Yildiz BO (2004). The prevalence and features of the polycystic ovary syndrome in an unselected population. J Clin Endocrinol Metab.

[6] Homburg R (2005). Clomiphene citrate--end of an era? A mini-review. Hum Reprod.

[7] Gysler M, March CM, Mishell DR Jr, Bailey EJ (1982). A decade's experience with an individualized clomiphene treatment regimen including its effect on the postcoital test. Fertil Steril.

[8] Teede HJ, Misso ML, Costello MF (2018). Recommendations from the international evidence-based guideline for the assessment and management of polycystic ovary syndrome. Hum Reprod.

[9] Atay V, Cam C, Muhcu M, Cam M, Karateke A (2006). Comparison of letrozole and clomiphene citrate in women with polycystic ovaries undergoing ovarian stimulation. J Int Med Res.

[10] (2004). Revised 2003 consensus on diagnostic criteria and long-term health risks related to polycystic ovary syndrome. Fertil Steril.

[11] Tocci A, Lucchini C (2010). WHO reference values for human semen. Hum Reprod Update.

[12] Bansal S, Goyal M, Sharma C, Shekhar S (2021). Letrozole versus clomiphene citrate for ovulation induction in anovulatory women with polycystic ovarian syndrome: a randomized controlled trial. Int J Gynaecol Obstet.

[13] Ibrahem MA (2019). Simultaneous letrozole and clomiphene citrate versus letrozole alone in clomiphene citrate resistant polycystic ovary syndrome: a randomised controlled trial. Open J Obstet Gynecol.

[14] Mejia RB, Summers KM, Kresowik JD, Van Voorhis BJ (2019). A randomized controlled trial of combination letrozole and clomiphene citrate or letrozole alone for ovulation induction in women with polycystic ovary syndrome. Fertil Steril.

[15] Hajishafiha M, Dehghan M, Kiarang N, Sadegh-Asadi N, Shayegh SN, Ghasemi-Rad M (2013). Combined letrozole and clomiphene versus letrozole and clomiphene alone in infertile patients with polycystic ovary syndrome. Drug Des Devel Ther.

